# Twelve years of repeated wild hog activity promotes population maintenance of an invasive clonal plant in a coastal dune ecosystem

**DOI:** 10.1002/ece3.2045

**Published:** 2016-03-15

**Authors:** Callie A. Oldfield, Jonathan P. Evans

**Affiliations:** ^1^ Department of BiologyThe University of the SouthSewaneeTennessee37383

**Keywords:** Disturbance, facilitation, geophyte, perennial, tuber, ungulate

## Abstract

Invasive animals can facilitate the success of invasive plant populations through disturbance. We examined the relationship between the repeated foraging disturbance of an invasive animal and the population maintenance of an invasive plant in a coastal dune ecosystem. We hypothesized that feral wild hog (*Sus scrofa*) populations repeatedly utilized tubers of the clonal perennial, yellow nutsedge (*Cyperus esculentus*) as a food source and evaluated whether hog activity promoted the long‐term maintenance of yellow nutsedge populations on St. Catherine's Island, Georgia, United States. Using generalized linear mixed models, we tested the effect of wild hog disturbance on permanent sites for yellow nutsedge culm density, tuber density, and percent cover of native plant species over a 12‐year period. We found that disturbance plots had a higher number of culms and tubers and a lower percentage of native live plant cover than undisturbed control plots. Wild hogs redisturbed the disturbed plots approximately every 5 years. Our research provides demographic evidence that repeated foraging disturbances by an invasive animal promote the long‐term population maintenance of an invasive clonal plant. Opportunistic facultative interactions such as we demonstrate in this study are likely to become more commonplace as greater numbers of introduced species are integrated into ecological communities around the world.

## Introduction

As invasive species are introduced into native communities around the world, they can facilitate the establishment of other invasive species (Richardson et al. 2000; Simberloff 2006). Simberloff and Von Holle (1999) coined the term “invasional meltdown” to describe the process by which this facilitation leads to the increased success and further establishment of invasive species and an eventual shift in community composition (Simberloff 2006). Examples of such positive invader–invader interactions among plants and animals fall into two basic categories: (1) pollination/dispersal such as by an invasive pollinator improving the reproduction of an invasive plant, or an invasive animal preferentially eating and dispersing the seeds of an invasive plant (Simberloff and Von Holle 1999; Richardson et al. 2000; Barthell et al. 2001; Goulson 2003; Traveset and Richardson 2006; Aizen et al. 2008; Lafleur et al. 2009; Nuñez et al. 2013; Beavon and Kelly 2015; Prior et al. 2015) and (2) disturbance whereby invasive animals alter habitat so that it favors invasive plants (Hobbs and Huenneke 1992; Kotanen 1995; Lozon and MacIsaac 1997; Schiffman 1997; Simberloff and Von Holle 1999; DiTomaso 2000; Vavra et al. 2007; Knight et al. 2009). Many examples of facilitation through disturbance involve feral animals, particularly the invasive non‐native *Sus scrofa* Linnaeus, 1758 (wild hog) (Spatz and Mueller‐Dombois 1972; Stone 1985; Loope et al. 1988; Vavra et al. 2007; Walter and Levin 2008; Spear and Chown 2009; Firn et al. 2010). Wild hog foraging disturbances, known as rooting, have generally been shown to facilitate invasive plants (Spatz and Mueller‐Dombois 1972; Cushman et al. 2004; Tierney and Cushman 2006; Fujinuma and Harrison 2012; Barrios‐Garcia and Simberloff 2013); some examples of plants species positively associated with wild hog disturbance in the United States include *Myrica faya* (Stone and Taylor 1984; Simberloff and Von Holle 1999), *Aira caryophyllea* (Kotanen 1995), *Sapium sebiferum* (Siemann et al. 2009), and *Holcus lanatus* (Spatz and Mueller‐Dombois 1972).

The novel interactions associated with invasional meltdowns involve facilitative processes between species that promote population maintenance over time (Gurevitch 2006). While many have posited the existence of such novel interactions, there have been few studies that provide the kind of demographic evidence of how such facilitative processes may be beneficial at the population level (Gurevitch 2006; Simberloff 2006). It has been suggested that invasive wild hog foraging disturbance may facilitate and be encouraged by particular invasive plant species (Stone 1985; Loope et al. 1988). Our study examined the maintenance of *Cyperus esculentus* L. (yellow nutsedge) populations over 12 years as a function of hog foraging disturbance. Clonal perennial yellow nutsedge, a highly successful invasive plant, can be found throughout the contiguous United States (Bendixen and Nandihalli 1987; Duncan and Duncan 1987). In the southeastern United States, yellow nutsedge tubers are a preferred food of invasive wild hogs, which will dig up large areas of soil to unearth the tubers (Wood and Roark 1980; Graves 1984; McAlister and McAlister 1993). In order to examine the existence of a potential facilitative relationship between yellow nutsedge and wild hogs, we tested the following hypotheses: (1) Yellow nutsedge culm and tuber densities are higher in hog disturbed areas, and disturbed areas have a lower percent cover of native plant species and (2) yellow nutsedge maintains higher densities in hog disturbed areas over a multiyear period as a result of repeated hog visitation to the same sites.

## Methods

### Species description

Yellow nutsedge is an invasive clonal perennial that is disturbance‐dependent (Bendixen and Nandihalli 1987; Duncan and Duncan 1987; Renne and Tracy 2007). The sedge is thought to originate from Africa and Europe, and it has been cultivated as food by humans from ancient Egypt to present‐day Africa and Europe (Linssen et al. 1989; Pascual et al. 2000; Defelice 2002). Yellow nutsedge is now considered a common weed throughout the United States and is classified as non‐native invasive in coastal Georgia (Bendixen and Nandihalli 1987; Zomlefer et al. 2008). Rhizomes grow vertically from tubers in the spring and generate a basal bulb at the soil surface. Basal bulbs produce shoots (culms) that include leaves, inflorescences, and roots. Culms also sprout horizontal rhizomes during the growing season, which generate new culms, forming large integrated clones (Wills et al. 1980; Stoller 1981). Throughout the growing season, but especially with shortened day length, rhizomes are stimulated to grow downward and terminate in tubers (Stoller 1981; Williams 1982). Yellow nutsedge is a prolific producer of tubers, with a single individual able to produce over 600 tubers in 4 months (Webster 2005). By winter, all culms have senesced, and a clone persists as a collection of disconnected tubers underground (Mulligan and Junkins 1976; Kelley 1990). These tubers can remain dormant for up to three and a half years depending on soil depth (Stoller and Wax 1973; Kelley and Fredrickson 1991). Tuber dormancy can be broken following soil disturbance (Kelley 1990; Kelley and Fredrickson 1991), but tuber production becomes severely limited when subjected to light competition with other plants (Stoller 1981; Stoller and Sweet 1987).

Wild hogs are native to Eurasia, but have been introduced to all continents except Antarctica (Graves 1984; Long 2003). Male wild hogs are generally solitary when not breeding, but females may form groups consisting of up to four females and their piglets (Graves 1984). Breeding is dependent on food availability and can continue throughout the year, with the most frequent breeding events occurring in the fall and winter (Graves 1984; Baber and Coblentz 1986); litters consist of approximately 5 piglets (Graves 1984; Baber and Coblentz 1986). North American predators on wild hogs include bears, wildcats, and alligators, although hunting is the main pressure on populations (Graves 1984). The diet of wild hogs in southeastern coastal ecosystems varies throughout the year, but is primarily made up of fruits, vegetation, and underground plant material (Wood and Roark 1980). Wild hogs use their snouts to forage for food under the soil surface (Graves 1984), creating distinctive patches of upturned soil and vegetation (Kotanen 1995). This disturbance typically affects the top 5 to 15 cm of the soil and can range in extent from one meter to one hectare sized patches, although smaller disturbances are more common (Kotanen 1995). These disturbances have been shown to disrupt natural communities and encourage the spread of invasive species (Aplet et al. 1991; Kotanen 1995; Tierney and Cushman 2006; Barrios‐Garcia and Simberloff 2013).

### Study area

Our study was conducted on St. Catherine's Island, a 5600 ha barrier island located on the coast of Georgia, USA. Like many barrier islands along the southeastern coast, St. Catherine's Island has a long history of prehistoric and historic land use (Thomas et al. 1978; Jones and Coile 1988). Prior to 1860, half of the forests on the island were converted to agricultural fields for the purpose of growing Sea Island cotton (*Gossypium barbadense* L.). The vegetation has also been logged, burned, and used for grazing animals. Plant communities on the island have been transformed by mammalian herbivore activity (Thomas et al. 1978). Between 1945 and 1975, the island was used for extensive cattle ranching (Thomas et al. 1978; Jones and Coile 1988). More recently, extensive browse by white‐tailed deer (*Odocoileus virginianus* Zimmerman, 1780) has caused regeneration failure within woody plant communities (Evans and Keen 2013). Since 2005, forests on the island have been further altered by the loss of a dominant woody species, redbay (*Persea borbonia* (L.) Spreng.), as a result of the spread of laurel wilt by an invasive pathogen‐carrying insect, the redbay ambrosia beetle (*Xyleborus glabratus* Eichhoff, 1877) (Koch and Smith 2008; Evans et al. 2014). Wild hogs were brought to the island in the 1930s as livestock (Thomas et al. 1978; Jones and Coile 1988) that subsequently became feral. Wild hogs have been associated with tree recruitment failure in maritime forests on St. Catherine's Island (Evans and Keen 2013).

On the east side of the island, there are dune ridge and swale systems undergoing early succession to woody plant communities (Thomas et al. 1978; Jones and Coile 1988). On coastal barrier islands including Florida, Georgia, and Texas, yellow nutsedge inhabits open swales (McAlister and McAlister 1993; J Evans personal observation), which are interdunal depressions characterized by more stable environmental conditions compared to surrounding dune ridge and strand communities (Miller et al. 2010). Newly formed swales occur as a result of shoreline accretion and overwash disturbance processes associated with the ocean side of the island (Hosier and Cleary 1977). Our study was conducted in two dune swale sites: one on the northeast corner of the island, known as the North Beach site (31°41.075″ N, 81°07.987″ W), and the other on the east‐facing side, known as the McQueen's Inlet site (31°37.695″ N, 81°07.987 ″W). The two sites are separated by forest, salt marsh, and inlets for a distance of 6.3 km. Dune swale vegetation at these sites was characterized by a mixture of native perennial and annual grasses (i.e., *Spartina patens* (Aiton) Muhl, *Panicum amarum* Elliott, *Cenchrus* L. sp.), sedges (i.e., *Fimbristylis* Vahl sp., *Scirpus* L. spp.), and forbs (i.e., *Hydrocotyle bonariensis* Lam., *Croton punctatus* Jacq., *Phyla nodiflora* (L.) Greene, *Sabatia stellaris* Pursh), along with isolated clumps of the shrub *Morella cerifera* (L.) Small (J. Evans unpubl. data; Jones and Coile 1988).

### Field methods

In 1997, a 0.2 ha area at each study site was surveyed for wild hog rooting disturbances. Each disturbance was mapped and designated as a “disturbed plot” (North Beach: *n* = 4; McQueen's Inlet: *n* = 6). Adjacent to the “disturbed plots,” we established “undisturbed plots” which were control plots of comparable size and elevation. Hog disturbances reoccurred in disturbed plots in 2002 and 2008. In years of disturbance (1997, 2002, 2008), we randomly subsampled the following variables in each of the paired plots using 0.01 m^2^ quadrats: yellow nutsedge culm density, yellow nutsedge tuber density (2002 and 2008 only), and percent live cover of native species. The number of subsamples varied with size of disturbance, and data were averaged so that there was one value per disturbance. Tubers were extracted from the middle of each random sample from an area of 225 cm^2^ to a depth of 25 cm. In 2008, six additional paired plots were added at North Beach (*n* = 10) and four at McQueen's Inlet (*n* = 10) to outline new wild hog disturbances. No wild hog disturbances were observed in undisturbed paired plots throughout the duration of the study. To track the disturbance patterns over time, yellow nutsedge culm density was randomly subsampled at McQueen's Inlet plots annually in June over a period of thirteen years. Culm density categories were assigned using the following classes (number per 0.1 m^2^): 0–2, 3–8, and >8.

### Data analysis

To evaluate the effect of year and wild hog disturbance on yellow nutsedge culm density and tuber density and percent live cover of native species, we used the library *lme4* (Ihaka and Gentleman 1996; Bates et al. 2015a) in R (R Development Core Team 2014) to fit generalized linear mixed models. As fixed effects, we used treatment (undisturbed or disturbed) and year; as random effects, we used beach site and replicate nested within each site. Results may vary by beach site due to the differing environmental conditions between the two sites; results may vary by replicate as a result of microclimate, which may consistently impact the growth of plants. Generalized linear mixed‐effects models of counts of culms and tubers were fit assuming an underlying Poisson distribution and log link function. Linear mixed‐effects models of percent cover were evaluated (Bates et al. 2015a). A Shapiro–Wilk test revealed a non‐normal distribution of the percent cover of yellow nutsedge (*W* = 0.97, *P* = 0.041), so arcsine transformations were applied. After transformations, residual plots were approximately normally distributed and did not reveal deviations from homoscedasticity. Generalized linear mixed models were fit using the Laplace approximation, and the linear mixed‐effects model was fit using residual maximum likelihood (Bates et al. 2015b). For the mixed‐effects models, we used the Satterthwaite approximation to estimate the degrees of freedom of our models (Kuznetsova et al. 2015). For the generalized mixed‐effects models, we first assessed the dispersion of our models using the ratio of the Pearson chi‐square to the residual degrees of freedom (Bolker et al. 2009). If the data was overdispersed, we implemented the Wald t‐test that was also used for the linear mixed‐effects model (Bolker et al. 2009) and included a random effect of observation number (Harrison 2014). Effect sizes from the generalized linear mixed models were back‐transformed to account for the log link function.

## Results

### Density of culms and tubers in disturbed and undisturbed plots

Counts of culm and tuber abundance indicated overdispersion. The ratio of the Pearson chi‐square and residual degrees of freedom resulted in a ratio of 1.92 for culms and 4.25 for tubers. Variance associated with the random effects in culm, tuber, and native cover models was small and generally overlapped zero (Table 1).

**Table 1:** Summary of the random effects of replicates within sites and beach sites

Model	Variance	Standard deviation
Culms		
Replicate	<0.001	<0.001
Site	<0.001	<0.001
Observation	0.406	0.637
Tubers		
Replicate	<0.001	<0.001
Site	<0.001	<0.001
Observation	3.6	1.987
Live cover		
Replicate	<0.001	<0.001
Site	0.046	0.214

**Figure 1:**
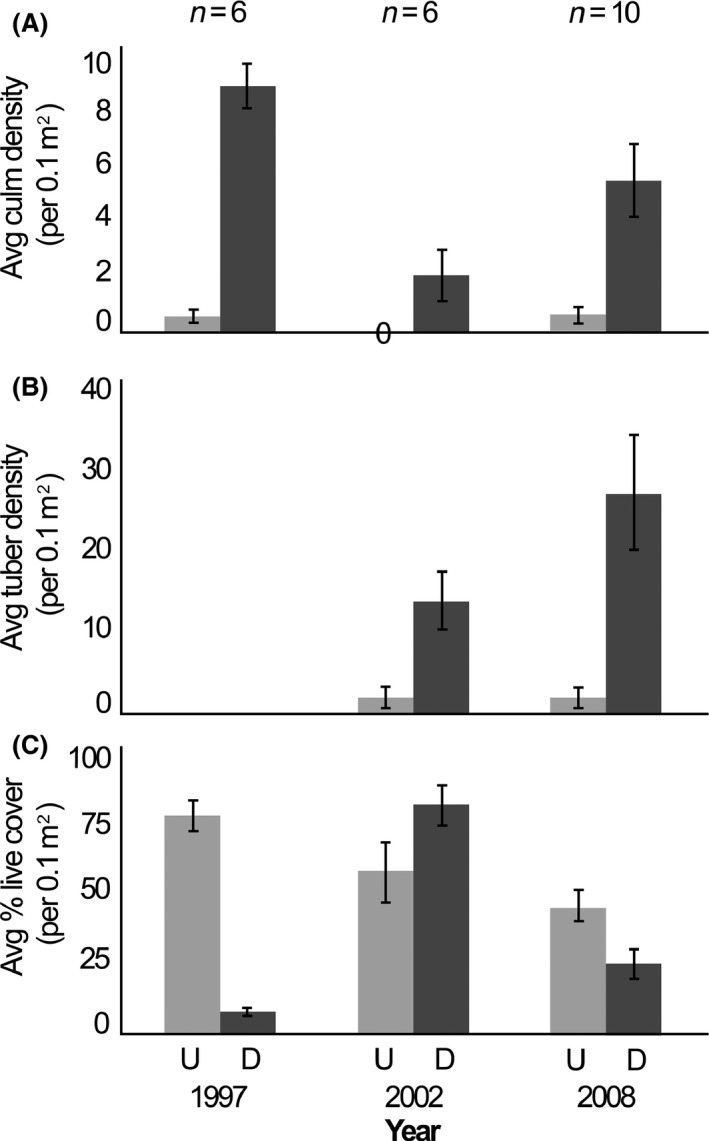
Mean number of yellow nutsedge (*Cyperus esculentus* L.) culms (A), mean yellow nutsedge tuber density (B), and mean percent native cover (C) ± SE per 0.1 m^2^ of undisturbed and disturbed plots at McQueen's Inlet. *U* represents undisturbed control plots, and *D* represents disturbed plots.

**Figure 2:**
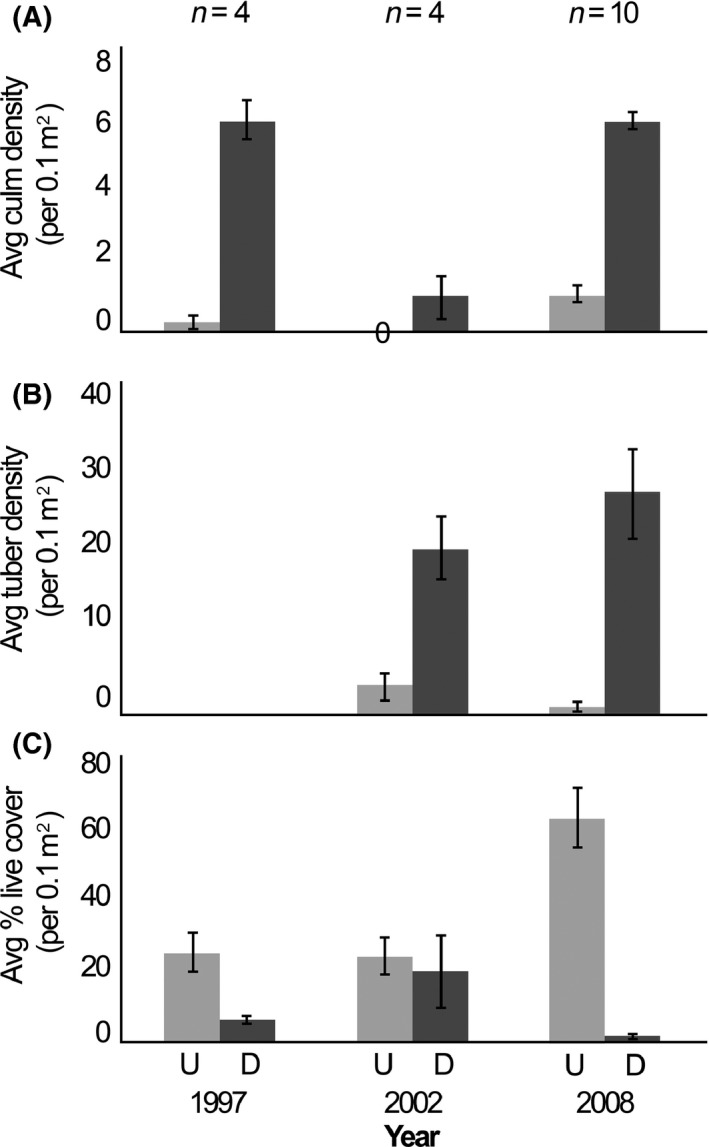
Mean number of yellow nutsedge (*Cyperus esculentus* L.) culms (A), mean yellow nutsedge tuber density (B), and mean percent native cover (C) ± SE per 0.1 m^2^ of undisturbed and disturbed plots at North Beach. *U* represents undisturbed control plots, and *D* represents disturbed plots.

After controlling for the effects of replicate and site, we found significantly more yellow nutsedge culms in the disturbed plots compared to the undisturbed. On average, disturbed sites had 3.6 more culms than undisturbed sites (95% CI: 2.25–4.93; *t*‐value: 5.26, residual df = 73, *P* < 0.001). No variation associated with year was detected (*t*‐value: 2.20, residual df = 73, *P* = 0.028; Figs. 1a and 2a).

Tuber density trends confirmed that tubers were present before and after wild hog disturbance events in disturbed areas, but in differing densities. Disturbed plots had 2.5 more tubers than undisturbed plots (95% CI: 0.47–4.56), even directly after disturbance years (*t*‐value: 2.41; residual df = 73, *P* = 0.016). Year had no significant effect on tuber density (t‐value: 0.98, residual df = 73, *P* = 0.33; Figs. 1b and 2b).

On average, there was 39.3% less live cover of native species in disturbed plots compared to undisturbed plots after controlling for site and replicate (95% CI: 31.8–46.8%; *t*‐value: −5.25; df: 76, *P* < 0.001). Even though year had no effect in our model, 2002 preceded a wild hog disturbance event where we observed live cover of native species to be 25% higher at the McQueen's Inlet disturbed plots (*t*‐value: −0.34; df = 76, *P* = 0.74; Figs. 1c and 2c).

### Nutsedge population maintenance and hog disturbance frequency

**Figure 3:**
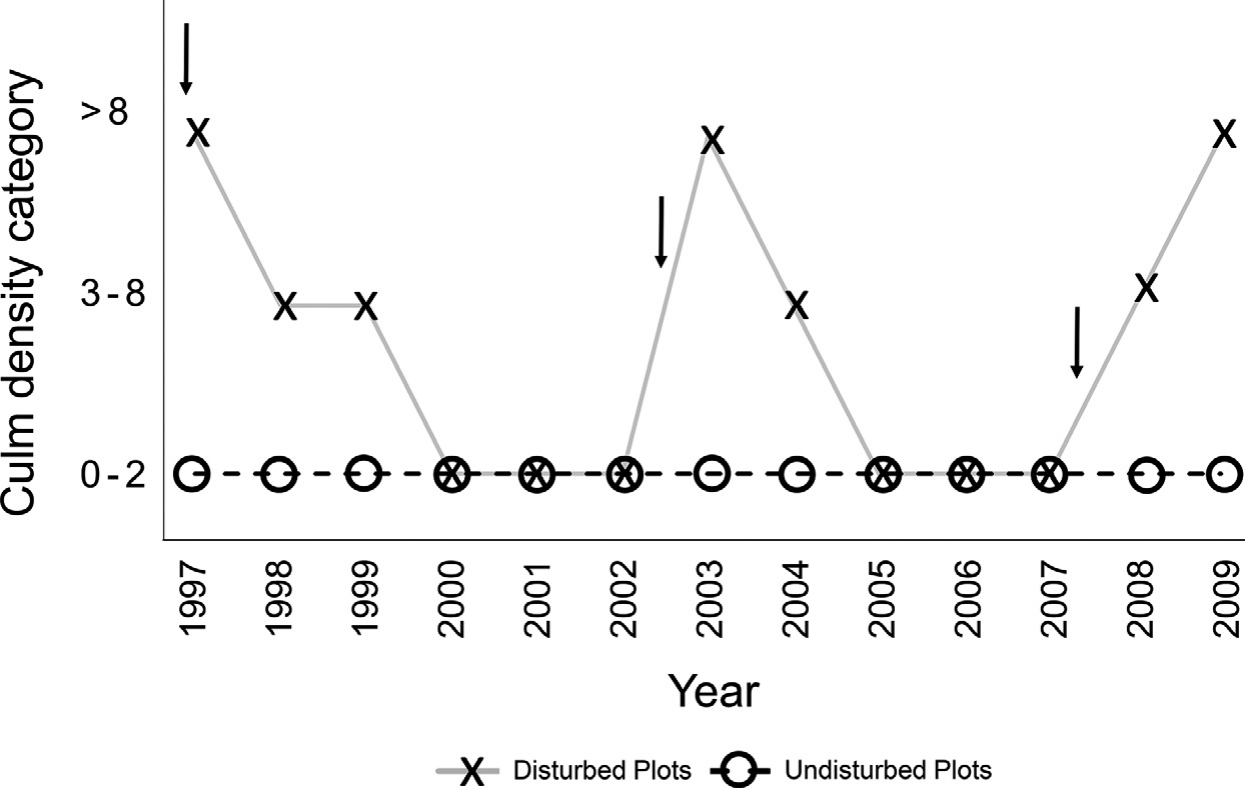
Data showing yellow nutsedge (*Cyperus esculentus* L.) culm density at McQueen's Inlet comparing disturbed to undisturbed plots. Categories are defined as follows: 0–2, 3–8, and ›8 culms per 0.1 m^2^. Over the course of the 13‐year period, disturbed plots experienced hog activity on three separate occasions as indicated by arrows.

Disturbance areas at McQueen's Inlet and North Beach were observed each year from 1997 to 2009, revealing a pattern of regular wild hog disturbance events. Wild hogs disturbed sites in 1997, 2003, and 2008, roughly every 5 years. The exact timing of the disturbance events between yearly samples is not known. Annual trends of culm density repeatedly showed a decrease in yellow nutsedge culms preceding a disturbance event and an increase following disturbance. Yellow nutsedge culm density in undisturbed plots was consistently in the 0‐2 culms per 0.1 m^2^ category, whereas yellow nutsedge culm density in disturbed plots fluctuated between having 0‐2 to over 8 culms per 0.1 m^2^ depending on the timing of the last disturbance (Fig. 3).

## Discussion

We found a significant positive association between rooting disturbances by invasive wild hog and the maintenance of invasive yellow nutsedge populations on St. Catherine's Island. Over the 12‐year period, culm and tuber density was consistently higher in disturbed plots compared to undisturbed plots, supporting our hypotheses that wild hog rooting behavior facilitated the maintenance of the yellow nutsedge populations. Wild hogs returned to the disturbed plots approximately every 5 years and uprooted and eliminated plant cover of native species. As a result, this disturbance promoted the vegetative growth of the remaining uneaten yellow nutsedge tubers. The presence of recently dead culms in wild hog rooting sites suggested that disturbances occurred prior to the June sampling in that growing season. While there is a clear pattern of site fidelity by wild hog populations, the actual frequency may dependent on a number of factors associated with the rate at which the yellow nutsedge populations rebound following disturbance, including variable water availability during the growing season (Bendixen and Nandihalli 1987) and rate of recolonization by native species. Our results show that tuber density was an order of magnitude greater than culm density in disturbed sites. The yellow nutsedge population was reestablished initially from remaining unconsumed tubers that produced culms that spread rhizomatously, increasing culm density throughout disturbed areas. In the first year postdisturbance, the population was most likely represented more by culms rather than tubers. Our data suggest that, in subsequent years, the tuber density rebounds, with some tubers contributing to the next year's culm production, while others remain dormant. The increased tuber density over time replenishes the food crop for the wild hog population.

While several studies have shown that wild hogs can facilitate the establishment of invasive plant species (Aplet et al. 1991; Kotanen 1995; Tierney and Cushman 2006; Barrios‐Garcia and Simberloff 2013), these studies did not observe or did not consider the importance of repeated return intervals by wild hogs. Our long‐term data provide evidence of such facilitation as shown by the repeated return interval to study sites and a positive density response to the hog disturbance by yellow nutsedge. Tardiff and Stanford (1998) document a similar positive interaction involving native species. They found that grizzly bears (*Ursus arctos horribilis* Ord. 1815) in Montana create soil disturbances as they dig up glacier lily (*Erythronium grandiflorum* Pursh) bulbs as food (Tardiff and Stanford 1998). Grizzly bear foraging disturbances are thought to be ecologically equivalent to wild hog disturbances (Work 1993; Tardiff and Stanford 1998; Sweitzer and Van Vuren 2002; Palacio et al. 2013). The grizzly bears miss some bulbs, which allow the glacier lily to recolonize postdisturbance. The authors speculated that increased nutrient content of the geophytes in these disturbances may be the reason grizzly bears revisit the sites, resulting in positive feedback (Tardiff and Stanford 1998).

Wild hog rooting disturbance in coastal systems can be characterized as high intensity, but low frequency and localized spatial extent (Kotanen 1995). The uprooting and consumption associated with wild hog rooting activity results in major reduction in vegetative cover and the disruption of soil processes. Wild hog activity creates small patches of early successional conditions. Early successional dunes have been shown to be characterized by limited nitrogen availability that is highly patchy in its distribution. Cain et al. (1999) suggest that such heterogeneous resource availability patterns select for spreading rhizomatous species such as yellow nutsedge. Traits such as foraging and physiological integration allow rhizomatous clonal plants to maintain extensive clones in early successional dunes and contribute to their success in these systems (Evans 1991; Evans and Cain 1995). Our results show that clonal growth generated from unconsumed tubers, rather than seed dispersal and seedling recruitment, promotes the long‐term maintenance of yellow nutsedge populations in response to wild hog disturbance. Similar results were found by Kotanen (1995) where certain species were able to grow vegetatively in the disturbance for up to a year before competing with other species that were established through seed, and it was found that some geophytes remain in high densities, despite being preferred food. However, other studies document the importance of seedling establishment in wild hog disturbances (Kotanen 1995; Cushman et al. 2004; Tierney and Cushman 2006; Barrios‐Garcia and Simberloff 2013).

Simberloff (2006) and Gurevitch (2006) point to the lack of demographic evidence in studies that purport facilitation between invasive species. Our documentation of a clear population‐level benefit for yellow nutsedge as a result of repeated hog disturbance fulfills this expectation. However, we recognize that in order to provide evidence for an invader–invader mutualism, a population‐level benefit must be documented for both partners (Simberloff 2006). In our case, this would be very difficult because wild hogs on islands along the coast of Georgia are subject to population management (Jones and Coile 1988; Hamrick et al., 2011), and given the omnivorous behavior of hogs, it would be hard to isolate the population‐level consequences of a single food source without experimental manipulation. However, due to the nutritive nature of this relationship and repeated visitation of sites, it can be expected that wild hogs benefit from the consumption of yellow nutsedge tubers. Not only are yellow nutsedge tubers a preferred food of wild hogs (Wood and Roark 1980; Graves 1984; McAlister and McAlister 1993), but Palacio et al. (2013) show that wild hog rooting increases the size and nutrient content in remaining geophytes, as was the case in the previously mentioned grizzly bear‐glacier lily example (Tardiff and Stanford 1998). This may provide reason for the return interval of wild hog visits to previously rooted sites (Palacio et al. 2013).

Invader–invader or invader–native positive interactions involving plants can be formed in a variety of ways (Traveset and Richardson 2011, 2014). Coevolved invasive mutualists may invade a new environment together such as seen in invasive figs (*Ficus* L. spp.) and their wasp mutualists in New Zealand (Gardner and Early 1996). An invasive species may act as a surrogate for an ecologically similar native partner and facilitate seed dispersal or pollination (Goulson 2003; Traveset and Richardson 2006, 2011; Mondor and Addicott 2007; Aizen et al. 2008; Burns 2012). More rarely, species may form novel interactions for which there has been no previous partner (Richardson et al. 2000; Traveset and Richardson 2011). Richardson et al. (2000) describes an example of this type of interaction that has recently formed between an invasive wind‐dispersed pine and a bird that now disperses its seeds in Australia. We believe our study represents another example of this type of interaction. Yellow nutsedge and wild hogs have formed an opportunistic facultative interaction in a new environment. It is difficult to predict where these new interactions will form (Richardson et al. 2000), but they will undoubtedly become more common as greater numbers of invasive species integrate into native communities in the future.

There are numerous examples of positive interactions in which one partner promotes and protects the other as a food source (Farji‐Brener and Illes 2000; Mueller and Gerardo 2002; Hata and Kato 2004; Brock et al. 2011; Pion et al. 2013; Casey et al. 2014). These have been termed “farming” mutualisms. Farming mutualisms are either high‐level, where the animal and crop have coevolved such that the animal plants, fertilizes, and defends crops, or low‐level, where animals simply disturb ecosystems through foraging activity, resulting in the promotion of a species that is suited to thrive in the disturbance (Silliman and Newell 2003). Recently, invader–native mutualisms between ants and honeydew‐producing hemipterans have highlighted the fact that these farming interactions need not be obligate or between native species (Lach 2007; Mondor and Addicott 2007; Styrsky and Eubanks 2010). In this case, the ants acted as a surrogate (preadapted) for a native mutualist (Mondor and Addicott 2007). Because the act of harvesting yellow nutsedge is also a form of tending, wild hog behavior in its relationship to the yellow nutsedge population can be thought of as an invader–invader example of low‐level farming as defined by Silliman and Newell (2003).

This is not the first instance of the promotion and harvesting of yellow nutsedge by an animal. Yellow nutsedge was one of the first agricultural crops domesticated by humans, and a variant (*C. esculentus* var. *sativus* Boeckl.) is still grown around the world today both for human and livestock (including wild hogs) consumption (Pascual et al. 2000; Defelice 2002). The utilization of yellow nutsedge in an ancient agricultural context is likely related to the fact that it is an easily propagated clonal species (Defelice 2002). This clonal life history is also taken advantage of by wild hogs. In summary, the act of wild hogs rooting for yellow nutsedge tubers results in an incomplete harvest, where some tubers are eaten and others remain in the disturbed environment. The soil disturbance by wild hogs serves the dual function of eliminating other live plant cover and reestablishing the perpetuation of future yellow nutsedge crops. Our study provides demographic evidence of how the activity of one invasive species can promote the population maintenance of another invasive species. Opportunistic facultative interactions such as demonstrated here are likely to become more commonplace as greater numbers of introduced species are integrated into ecological communities around the world.

## Conflict of Interest

None declared.
